# Animal-derived ingredients in medicines: a framework for ethical prescribing practices

**DOI:** 10.3389/fphar.2025.1693059

**Published:** 2025-09-29

**Authors:** Hamdi Lababidi, Christopher Bobier, Daniel Rodger

**Affiliations:** ^1^ Foundational Sciences, Central Michigan University College of Medicine, Mt Pleasant, United States; ^2^ Foundational Sciences, London South Bank University, London, United Kingdom

**Keywords:** ethics, patient preference, prescription, animals, values and beliefs

## Abstract

An increasing number of patients want to avoid animal-derived ingredients in medications due to ethical, religious, or cultural beliefs, yet transparency about these ingredients remains limited. Healthcare professionals often lack guidance on how to respect their preferences while ensuring effective treatment. Here, we propose an ethical pharmacotherapy modification framework that guides clinicians through a stepwise approach, altering manufacturer, dosage form, administration method, or medication itself, to accommodate patient values without compromising clinical efficacy. This framework balances respect for patient autonomy with clinical beneficence. Implementing this approach is likely to enhance patient trust and adherence by aligning pharmaceutical care with individual ethical considerations and promoting patient-centered care.

## Introduction

A key tenet of patient-centered care is that healthcare providers consider individual patient values, beliefs, and preferences in their care (Epstein and Street, 2011). This requires engaging with patients, involving them in the decision-making process, and ensuring that they have any necessary information to make an informed choice about their care. Yet, the healthcare industry has lagged when it comes to transparency in medication ingredients and taking action to address patient preferences (Wang et al., 2025; Tieu et al., 2024). This matters because an increasing number of patients prefer to avoid animal-derived ingredients. In the United States alone, there are over 19.6 million vegans and vegetarians and over 17.8 million people with religious preferences related to medication ingredients (Pill Clarity, 2025). In the United Kingdom, 5% of the population adopt a vegan or vegetarian diet (Vegsoc, 2025) and over 4 million Hindus and Muslims have a religious preference related to avoid animal-derived ingredients (Rodger, 2022). Worldwide, the number of vegetarians and vegans is increasing year-over-year (Olsborn, 2025). In addition, Alpha-gal Syndrome, a disease that requires the avoidance of even minute quantities of red meat to prevent life-threatening allergic reactions, is estimated to affect around 450,000 people in the United States (Centers for Disease Control and Prevention, 2025). Requests for pharmaceutical without animal-derived ingredients are increasingly common (Wang et al., 2025).

Patient-centered care concerning animal preferences poses a number of clinical and practical questions. Should clinicians solicit patient preferences or does the responsibility for disclosing animal preferences fall primarily on patients? How, if at all, should conversations regarding animal preferences take place in a clinical setting, or do time constraints on healthcare personnel suggest a more suitable alternative would be the development of educational information? How should medical and pharmacy education better prepare healthcare personnel for these kinds of questions? This paper focuses on the following question: how should healthcare professionals respond when it is known that a patient has a preference to avoid animal-derived ingredients? This is important, for there is no standardized ethical guidance on how providers should respond when it is known that a patient would like to avoid pharmaceuticals with animal-derived ingredients.

This article offers an ethical framework for pharmacotherapy modification, one that respects patient preferences while maintaining an acceptable risk-benefit ratio for patients. After presenting clinical and ethical considerations that support the need for such a framework, we present a multi-step algorithm to assist clinicians in meeting their ethical responsibility to respect patient autonomy while ensuring clinical beneficence. The strengths and weaknesses of each step are discussed.

### Clinical considerations

Several clinical considerations should be kept in mind regarding animal-derived ingredients in medication. First, in the United Kingdom, an audit of the 100 most commonly prescribed primary-care medicines found that 74% contained at least one of the following excipients: lactose, gelatin, or magnesium stearate, and 20% contained gelatin (Tatham and Patel, 2014). Excipients and disclosure practices differ across jurisdictions, so the proportion may vary outside the United Kingdom; although limited research has examined the prevalence of animal-derived ingredients in pharmaceuticals across different settings, it is well recognized that many medicines contain such ingredients (Rodger and Blackshaw, 2019; Babos et al., 2021; Harding et al., 2023; Strickland, 2014). Notable examples include *heparin*, an anticoagulant sourced from porcine intestinal mucosa; *propofol*, an anesthetic drug formulated with egg components; *shellac*, derived from the lac insect and used in tablet coatings; *glycerin*, which may be sourced from animal fats; *gelatin*, a common ingredient in capsules often derived from bovine or porcine collagen; and *lanolin*, extracted from sheep’s wool and used in topical preparations.

Second, there is widespread lack of awareness among healthcare professionals regarding the presence of animal-derived materials in pharmaceutical products (Tatham and Patel, 2014). Empirical data suggest that a significant proportion of physicians and pharmacists are not aware of the extent to which commonly prescribed medications contain ingredients of animal origin (Wang et al., 2025). One survey found that approximately 70% of physicians were unaware that some medications include ingredients derived from pork and/or beef (Sattar et al., 2004). Similarly, a study focusing on urologists revealed that nearly 75% were not sure if urological medications contain gelatin. (Warburton et al., 2010). This knowledge gap is attributable, in part, to the fact that the clinical and ethical implications of animal-derived excipients are not routinely addressed within standard medical or pharmacy curricula (Hanna et al., 2021).

Third, it is not common practice for clinicians to ask patients about their preferences or restrictions concerning animal-derived materials in pharmaceuticals, despite the potential for ethical, cultural, or religious conflicts (Misko and Fox, 2022). Research shows that healthcare professionals rarely engage patients in discussions about their religious or spiritual beliefs, and as a result, religious, cultural, and dietary considerations are frequently overlooked in the context of prescribing (Harding et al., 2023). In fact, one survey reported that as few as 2% of doctors regularly inquire about their patients’ religion and spirituality (Abdulla et al., 2019). In another survey of 500 urological patients, 40% reported dietary restrictions that included avoidance of animal-derived substances, yet nearly half of this subgroup had been prescribed medications containing gelatin—a product derived from animal collagen (Strickland, 2014).

Fourth, even when patient preferences regarding the use of non-animal derived pharmaceuticals are clearly expressed, many clinicians report feeling inadequately prepared to respond effectively (Hanna et al., 2021; Wang et al., 2025; Daher et al., 2015). This lack of preparedness includes uncertainty about appropriate clinical alternatives and limited awareness of available resources or guidance for identifying medications that align with religious or ethical requirements, such as Halal-certified or vegan formulations. For example, one survey indicated that over 80% of pharmacists do not know where to access information about Halal medication alternatives, underscoring a significant gap in professional training and infrastructure (Butler et al., 2018).

### Ethical considerations

A healthcare professional’s approach to addressing (or failure to address) patient preferences regarding animal-derived ingredients in medications can have significant implications for patient care (Rodger and Blackshaw, 2019; Rodger, 2022). First, a failure to consider a clear patient preference to avoid or mitigate animal-derived ingredients in medication disrespects patient autonomy. A core tenet of medical ethics is respect for patient autonomy, and upholding patient autonomy requires that patients understand and are able to decide based on their values and preferences. For example, if a healthcare professional knows a patient is vegan or Muslim and does not disclose that a recommended medication contains animal-derived ingredients, the patient is denied the opportunity to make an informed choice. Failure to provide this information undermines autonomy, particularly when disclosure might have led the patient to refuse the medication or request an alternative treatment.

Second, patients are harmed by the psychological distress of having unknowingly ingested animal ingredients, thereby violating deeply held ethical, religious, or philosophical convictions. This violation can erode the patient-clinician relationship. Patients may perceive their healthcare provider as either unconcerned with their deeply personal values or, worse, incompetent in navigating critical aspects of patient-centered care. Such a breach of trust is not merely an abstract ethical failing; it is a preventable form of patient harm. This resulting distrust can increase the likelihood of medication non-adherence and facilitate harm, as patients, feeling betrayed or morally compromised, may discontinue vital treatments, leading to suboptimal health outcomes, disease progression, or even adverse events.

Finally, religiously and culturally competent care would seem to involve the solicitation of patient preferences regarding animal-derived ingredients. Just as healthcare institutions routinely inquire about and accommodate patient dietary restrictions for meals, members of the care team should, arguably, extend this same level of sensitivity and respect to pharmaceutical choices. This is most clearly applicable to patients for whom a healthcare professional has compelling reason to think they have deeply held preferences. To disregard deeply personal beliefs regarding animals in the context of medication prescription, while accommodating them in food, creates an inconsistent standard of care. Integrating this inquiry into routine patient intake acknowledges the holistic nature of patient wellbeing, where spiritual and ethical values are as integral as physical health.

### Ethical pharmacotherapy modification framework

The clinical and ethical considerations underscore pressing questions regarding whether and how to solicit patient preferences regarding animal-derived ingredients in medications. We are interested in how healthcare professionals should respond when it is known that a patient has a preference to avoid animal-derived ingredients. Consider, for example, a patient who requests that a physician or pharmacist ensure that a prescribed medication does not contain animal-derived ingredients. Suppose the physician or pharmacist consults publicly accessible resources such as DailyMed (U.S. National Library of Medicine, 2025) or the Electronic Medicines Compendium (Datapharm Ltd., 2025) and determines that the prescribed medication does contain such ingredients. In that case, the appropriate course of action becomes ethically complex, and there is currently no guidance on how to proceed. In this situation, healthcare professionals must navigate two core ethical obligations: respecting patient autonomy and promoting clinical beneficence by maintaining an acceptable risk–benefit ratio in treatment. To assist clinicians in making decisions that are both ethically sensitive and therapeutically sound, we propose the Ethical Pharmacotherapy Modification (EPM) Framework, which is a structured, stepwise approach to medication modification (Table 1).

Step 1: Manufacturer change

**TABLE 1:** EPM framework.

Step	Strategy	Example	Strengths	Limitations
1	Manufacturer change	Different albuterol inhaler brands vary in the use of ethanol	Minimally disruptive and accessible via public resources	Availability depends on formularies, and may require pharmacist input
2	Dosage form change	Amoxicillin capsule (gelatin) to a tablet or suspension	Preserves active drug and route, and information is readily available	May affect palatability/adherence and requires prescriber approval
3	Administration method change	Opening capsule and mixing with food (amoxicillin)	Maintains medication identity, and is useful in shortages	Not always pharmacokinetically safe, and evidence is lacking for many drugs
4	Medication change	Porcine heparin to argatroban	Respects patient preferences and avoids objectionable excipients	Most disruptive; requires nuanced judgement, and risk of efficacy/safety trade-offs

The first and least disruptive step in the EPM Framework involves altering the manufacturer of a medication to avoid objectionable medication ingredients. While the active pharmaceutical ingredient remains unchanged, different manufacturers may use distinct formulations that vary in their inactive components. Public resources such as DailyMed (U.S. National Library of Medicine, 2025) and the Electronic Medicines Compendium (Datapharm Ltd., 2025) provide manufacturer-specific medication ingredients to allow healthcare professionals to differentiate between the contents of different manufacturers of the same medication. For instance, different manufacturers of albuterol inhalers may or may not use ethyl alcohol in their formulation. This strategy is efficient as information on formulation differences is relatively accessible and it preserves the medication’s identity, dosage form, and method of administration.

However, there are limitations to this step. Physicians and other non-pharmacist providers often lack access to real-time drug inventory lists and insurance formularies that determine the availability and coverage of specific manufacturers for their patients. Without a pharmacist’s consultation, this may limit options for patients. However, manufacturer changes frequently do not require physician approval, facilitating the provision of medication to patients in a timely manner via direct consultation with a pharmacist. The change, including rationale, should be documented in the patient’s electronic health record.

Step 2: Dosage form change

A manufacturer change may not be possible, and so, the second step in the EPM Framework involves altering the dosage form of a medication. A dosage form is the physical presentation of a medication, such as tablets vs. capsules, solutions vs. suspensions, suppositories vs. enemas, and so forth. Certain dosage forms—particularly capsules—are more likely to contain animal-derived gelatin, making them incompatible with the preferences of many patients. Changing the dosage form allows the healthcare provider to maintain the same active ingredient and method of administration while eliminating objectionable components. For example, amoxicillin in the form of gelatin-containing capsules can be substituted for amoxicillin tablets or suspensions. Alteration of dosage form preserves both the method of administration and the pharmacologic identity of the medication. As with manufacturer changes, information about dosage form options is easily accessible through standard clinical references.

However, this strategy may present new challenges; for instance, switching from a flavor-masking capsule to a tablet may affect patient adherence due to changes in palatability, particularly in pediatric populations. Additionally, alterations in dosage form must be approved by the prescriber, which may prolong the dispensing of an alternative dosage form if the prescriber is not readily available. Different dosage forms also vary in cost and availability, thereby affecting patient access. Dosage form changes should be evidence-based and documented in the patient’s electronic health record.

Step 3: Method of administration change

The third step in the EPM Framework involves changing the method by which a medication is administered. This strategy may be particularly valuable when preferred dosage forms are unavailable due to shortages or when other modification strategies (e.g., manufacturer or dosage form changes) are unfeasible. For instance, when amoxicillin is indicated for treatment in a patient who expresses a preference to avoid animal-derived ingredients, and only amoxicillin capsules are available due to a shortage of other dosage forms, a clinician may instruct a patient to open the capsules and mix the capsule content with a soft food such as applesauce. This preserves the medication’s identity while circumventing the ingestion of gelatin found in many capsules.

However, this strategy carries practical and clinical limitations. It is not always clinically acceptable to modify the method of administration. For example, opening some capsules may result in unacceptable changes in pharmacokinetics or palatability, and this may pose a particular challenge for patients on chronic regimens. There are also medico-legal concerns if clinicians recommend administration modifications without sufficient supporting evidence or regulatory endorsement. While opening amoxicillin capsules is endorsed by the American Academy of Pediatrics (American Academy of Pediatrics, 2025), modifying the method of administration of other medications may lack evidence for clinical appropriateness. Patients must consult with a healthcare professional before altering any method of administration. Evidence-based clinical decision-making is especially necessary at this step to protect interests and should be documented in the patient’s electronic health record.

Step 4: Medication change

The final step in the EPM Framework involves substituting the medication itself with another that is also clinically appropriate but avoids or mitigates objectionable components. This approach may be considered a last resort and should only be pursued when modifying the manufacturer, dosage form, or method of administration is not feasible or does not align with patient values. In some cases—particularly when alternative medications are pharmacotherapeutically similar—this step may be preferable to altering the method of administration. This is because the latter may or may not pose particular difficulties with drug absorption, tolerability, or adherence. For example, for patients who wish to avoid porcine-derived heparin, a provider may consider prescribing argatroban, a synthetic anticoagulant, instead. While this substitution honors patient ethical or religious concerns, it requires nuanced clinical judgment, as clinical appropriateness must be carefully assessed to avoid unintended adverse outcomes. Providers must account for differences in efficacy, safety, pharmacokinetics, contraindications, and monitoring requirements, as well as individualize these decisions for different patients. This step may also demand a solid grasp of pharmacotherapeutics and access to appropriate clinical resources or pharmacist consultation. Although medication change is the most disruptive strategy in the framework, it reflects a strong commitment to patient-centered care when implemented carefully. As with the other steps, this step requires proper evidence of comparable efficacy and documentation in the patient’s electronic health record.

## Discussion

The EPM framework is intended to assist healthcare professionals in respecting patient choice while maintaining an ethical risk-benefit assessment. It is important to note that not all these modifications may be clinically appropriate even if a patient expresses a preference for an animal-free alternative; given the prevalence of animal-derived ingredients in pharmaceuticals, it may well happen that a suitable non-animal-derived alternative is unavailable. What is needed is a proper understanding of the patient’s perspective of this issue: some patients may follow a position to mitigate, rather than completely avoid, medications with animal-derived ingredients. Understanding this allows clinicians to make a good-faith effort to provide animal-free alternatives, which may be as suitable as the default animal-containing products. However, in the case of a lack of a suitable alternative, or if there is a genuinely increased risk of unacceptable efficacy, safety, or pharmacokinetics with the alternative, the clinician should explain this finding to the patient transparently, who may either agree to take the animal-containing product or decline treatment. Documentation of this scenario is crucial here to mitigate legal risks to the clinician’s practice.

This approach meets a much higher ethical, and arguably clinical, standard than to hide the issue of animal-containing medications from the patient: even if the approach leads to treatment refusal, the patient-clinician relationship is significantly enhanced and trust in clinicians can begin to rise again. By explicitly placing the choice in the patient’s hands, clinicians both honor individual moral convictions and strengthen the therapeutic alliance by demonstrating transparency and respect for patient agency. Such practice not only fosters trust but also helps align treatment with the patient’s broader conception of wellbeing, which extends beyond physiological outcomes to include ethical and spiritual integrity. This is crucial since surveys demonstrate that patient trust in medical doctors, pharmacists, and nurses have dropped precipitously (Saad, 2025).

Nonetheless, the EPM framework has limitations. First, it requires education and training that is often lacking in medical and pharmaceutical education (Hanna et al., 2021). While further research is needed to improve education and training on this topic, we offer the following recommendations to improve clinical instruction:

- The knowledge gap among current clinicians regarding this topic as well as its ethical importance should be highlighted to emphasize to learners the urgency of gaining competency in medication ingredient ethics.
- Clinical curricula should discuss various drug information resources available to clinicians to find information on medication ingredients.
- The above EPM Framework can be presented as an example of an algorithm to follow when navigating this issue.
- Objective structured clinical examinations of mock patients should make it routine for learners to probe patients for their dietary preferences, whether religious or otherwise, and explain that this is important to help navigate various medication ingredients in their treatments.

We call for further research to address this issue, since, without education and training, our framework remains aspirational. Second, the EPM framework is limited by the pharmaceutical marketplace. The framework highlights not only an ethical challenge within clinical practice but also a structural challenge for the pharmaceutical industry and regulators, whose innovation and policy decisions are integral to expanding the availability of acceptable alternatives.

## Data Availability

The original contributions presented in the study are included in the article/supplementary material, further inquiries can be directed to the corresponding author.
